# Variation in Sediment Available-Phosphorus in Dianchi Lake and Its Impacts on Algal Growth

**DOI:** 10.3390/ijerph192214689

**Published:** 2022-11-09

**Authors:** Yongchuan Chen, Qiao Chen, Degang Zhang, Li Tang

**Affiliations:** 1 College of Biological and Agricultural Sciences, Honghe University, Mengzi 661100, China; 2 College of Resources and Environment, Yunan Agricultural University, Kunming 650201, China

**Keywords:** available phosphorus, algal growth, Dianchi Lake, sediment, soluble-phosphorus, spatial and temporal dynamics

## Abstract

Sediment phosphorus (P) is an active component of the P cycle in lakes and its availability and movement could play an important role in eutrophication. Sediments from different depths in five representative sites of Dianchi Lake China, i.e., Haigeng, Dounan, Luojiacun, Xinjie, and Kunyang, were studied from January to December to evaluate the spatial and temporal dynamics in available-P and soluble-P and their impacts on algal growth. The results showed that the average sediment available-P and soluble-P were 41.2 mg kg^−1^ and 0.27 mg kg^−1^, respectively. Sediment available-P and soluble-P concentrations varied significantly among seasons, sites, and layers, with the following order: surface (0–5 cm) > middle (5–10 cm) > bottom (10–20 cm). The release and transformation characteristics of available-P and soluble-P at the sediment–water interface varied among sites. The Haigeng site, with the most severe algae blooms, had significantly higher average available-P and soluble-P in sediment than other sites. This indicated that phosphorus release and availability were associated with algal growth, and that the sediment surface (0–5 cm) is an important internal source that supports algal growth in Dianchi Lake.

## 1. Introduction

Phosphorus (P) is the main nutrient that regulates primary productivity in water bodies. Even when exogenous P is controlled, endogenous P released from sediments can sustain eutrophication in a water body [1]. Sediments can act as a large sink of phosphorus (P) at the lake bottom, but this reservoir of P can be remobilized back into the water through biogeochemical and physical processes [2,3], resulting in widespread lake eutrophication. Meanwhile, P in sediments can also determine the buffering capacity of sediments towards water P loads [4,5]. Sediment P accounts for 60%–80% of total-P loads in lakes and can maintain the P concentration equilibrium at the sediment–water interface of shallow lakes [1,6]. Phosphorus released from sediment can be an important source of nutrition, supporting algal growth in lakes [7,8,9]. P released by sediments can account for 30% of total-P input into a water body, reaching as high as 80% in some lakes and reservoirs [10]. Sediment available-P extracted by NaHCO_3_ has been used as a quantitative index for algae available-P [11,12]. Available-P is potentially mobile P, which contains partially dissolved phosphorus and loosely adsorbed P. Available-P of sediment is the most important factor determining P movement at the sediment–water interface, and its concentration and release are affected by many internal and external environmental factors, such as sediment properties, temperature, microbial community, and redox potential. Soluble-P is the most active and labile fraction of available-P in sediment and is easily released into water, so its concentration is not only directly related to the sediment available-P, but also the water-P concentration [13,14,15]. There have been reports on sediment P and its release, but most studies have not reflected the dynamic variability of P species in situ, especially in Dianchi Lake [16,17,18,19,20]. Dianchi Lake is a famous eutrophic lake in China which has had frequent cyanobacterial blooms for nearly four decades. Dianchi Lake is located to the southwest of Kunming city, China, and has an average area of 310 km^2^; additionally, the lake measures 40.4 km north to south and 7 km west to east and has 163.2 km of shoreline. In recent years, although the exogenous pollution in Dianchi Lake has been controlled, algal blooms still erupt from April to October every year, and the proliferation of harmful algae greatly damages the ecological function and landscape of the recreational waters. Therefore, endogenous P released from sediments plays an important role in algae growth in Dianchi Lake. Furthermore, previous field studies on sediment P concentrations in Dianchi Lake have only considered a single time point or a single site, not time series of sampling surveys over longer periods [21,22]. Some laboratory experiments have reported on P forms, as well as their loading and release behaviors [23,24]. The temporal and spatial distributions and variability of water-P and sediment total-P have been examined in Dianchi Lake [24,25,26]. However, there is very little information available on the release characteristics of various P forms at the sediment–water interface and their impacts on algal growth in lakes, especially in terms of the temporal and spatial dynamics of the release of available-P in Dianchi Lake when exogenous P is controlled. Due to the different origins of P and the ecological conditions in Dianchi Lake, P concentrations and availability in water and sediment varied greatly among different sites and seasons, as did algal growth, affected by nutrition and environmental conditions [25,26]. Therefore, it is necessary to study the release characteristics of various P forms at the sediment–water interface and their impacts on algal growth in Dianchi Lake. This paper studied the dynamic changes of available-P and soluble-P concentrations in sediment at different depths in five representative sites of Dianchi Lake to evaluate the spatial and temporal dynamics of sediment available-P and soluble-P, their release characteristics at the sediment–water interface, and their impacts on algal growth.

## 2. Materials and Methods

### 2.1. Sites and Sampling

**Figure 1:**
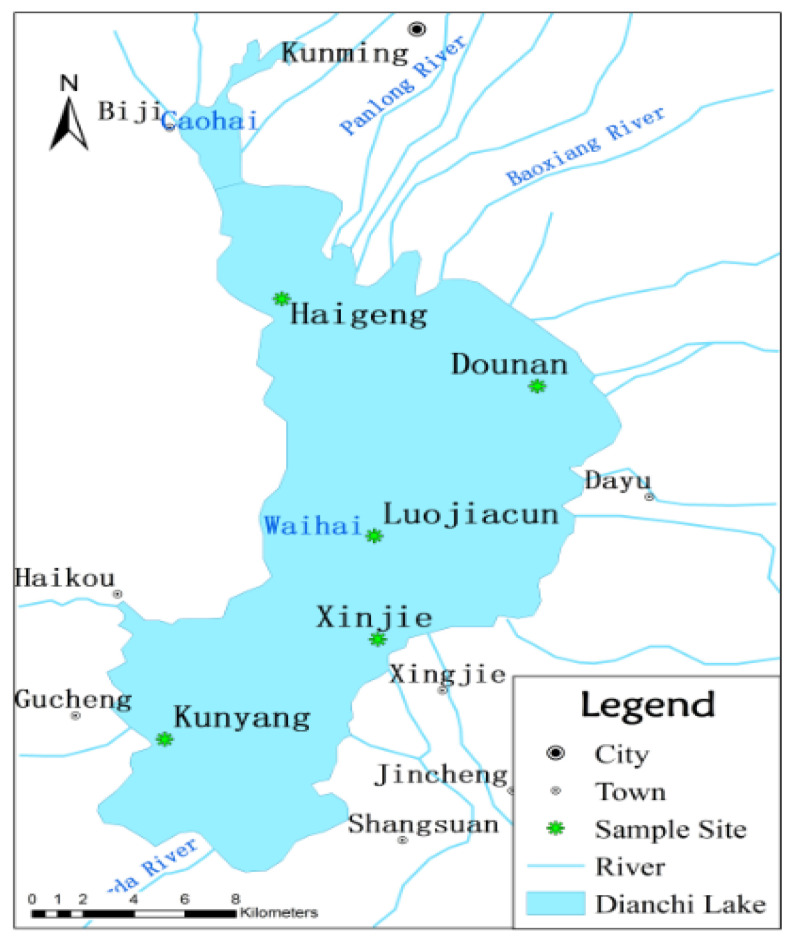
Sampling and monitoring sites in Dianchi Lake, China.

Dianchi Lake is located to the southwest of Kunming city (the capital and only large city of Yunnan Province, China) (Figure 1). Dianchi Lake (24°40′–25°02′ N, 102°36′–103°40′ E) is the largest lake in southwestern China and the sixth largest freshwater lake in China. The lake has an average area of 310 km^2^ and an average sea level of 1887.4 m; additionally, the lake measures 40.4 km north to south and 7 km west to east and has 163.2 km of shoreline. Moreover, the maximum and average water depths are 10.9 m and 4.4 m, respectively, and the watershed (Dianchi Lake basin) area is 2920 km^2^ [27]. Five representative sites were selected based on their geo-ecological locations and positioned with GPS in Dianchi Lake China (Figure 1) at the following locations: Haigeng (24°54.857′ N, 102°39.837′ E), Dounan (24°52.662′ N, 102°45.237′ E), Luojiacun (24°48.799′ N, 102°41.789′ E), Xinjie (24°46.157′ N, 102°41.868′ E), and Kunyang (24°43.599′ N, 102°37.362′ E).

The Haigeng site in Dianchi Lake is adjacent to Kunming city, where previous sediment-P has accumulated from large amounts of industrial wastewater and municipal sewage discharge. This is also the area with the most severe algae blooms in Dianchi Lake, and the water depth is 5 m. The Dounan site in Dianchi Lake is close to flower and vegetable production bases in Chenggong, so sediment P has been accumulated from P applied to flower and vegetable soils, and the water depth is 4 m. The Luojiacun site of Dianchi Lake is in the middle of Dianchi Lake and is far away from pollution sources, where the low water disturbance has facilitated the accumulation of sediment-P in the fine clay sediments, and the water depth is 7 m. The Xinjie site of Dianchi Lake is near rice production areas, where sediment P accumulation is mostly affected by agricultural activities and non-point pollution, and the water depth is 4 m. The Kunyang site of Dianchi Lake is near P fertilizer production bases, where sediment P accumulation in sediment comes from previous discharge of industrial wastewater containing high concentrations of P, and the water depth is 5 m.

Sediment and water samples were collected from the five representative sites every month from January to December 2018. Separate sediment samples were collected at 0–5 cm, 5–10 cm, and 10–20 cm depths with a Uwitec Core sampler (Austria) and air-dried for the determination of available-P (AP). Fresh sediment samples were used for the determination of soluble-P (SP) immediately after sampling. Water samples from each site were collected at three layers depending on site-specific water depths, i.e., surface layer, middle layer, and bottom layer, using an exhaust sampler (Institute of Hydrobiology, CAS). Water samples from each site were used for the determination of Chlorophyll-a concentration and water total-P immediately after sampling. The samples of the bottom water layer (about 10 cm above the sediment surface) were used to study the P concentration and release at the water–sediment interface. Three replicates were collected for each sample layer at each location. All collected sediment and water samples were immediately transported to the laboratory. A portion of the fresh sediment was used for determining soluble-P concentrations. The remaining sediment was air-dried and ground to determine available-P (1.0-mm sieve). A portion of each water sample was used for determining Chlorophyll-a concentration after filtering through a 0.45 μm cellulose membrane. The remaining water sample was used for determining water total-P.

### 2.2. Determination of Sediment-P, Water-P, and Chlorophyll-a

Concentrations of available-P (AP) in air-dried sediment samples were shake-extracted with 0.5 mol L^−1^ NaHCO_3_ (soil:liquid = 1:10) for half an hour and determined using the molybdenum blue-colorimetric method [12,28,29]. 

Fresh sediment samples were first shake-extracted with 0.01M CaCl_2_ (soil:liquid = 1:5) for half an hour. After filtration, soluble-P concentrations in the extracts were measured by the molybdenum blue-colorimetric method. Total-P concentration in water was determined using potassium persulphate and the molybdenum blue-colorimetric method. Chlorophyll-a concentration in water was determined using spectrophotometric colorimetric method via extraction with 80% acetone in low temperature 4 °C after filtering through a 0.45 μm cellulose membrane [13,14].

### 2.3. Statistical Analysis

Data (means ± standard deviation) were subjected to a one-way analysis of variance. Significant differences among sampling sites or sediment layers were compared by the least significant difference test at *p* < 0.05. The linear relationships among sediment P and water-P or sediment P and Chlorophyll-a were analyzed using Pearson’s correlation coefficient. All the data analyses were performed using SPSS 11.5 software (SPSS Inc., Chicago, IL, USA).

## 3. Results

### 3.1. Annual Variability in Sediment Available-P and Soluble-P in Dianchi Lake

Annual average sediment available-P concentrations in the five sites differed significantly among sediment layers (Table 1), with the highest concentration in the surface layer at 55.1 mg kg^−1^, followed by the middle layer at 41.1 mg kg^−1^, and the bottom layer at 27.3 mg kg^−1^. The coefficients of variation were similar among the three layers, ranging from 35% to 38%. The annual variation in sediment available-P was higher in the surface, ranging within 33.0–77.9 mg kg^−1^, compared with the middle and bottom layers, which had ranges of 25.6–56.6 mg kg^−1^ and 17.1–39.7 mg kg^−1^, respectively.

Annual variation in average soluble-P concentrations at the five representative sites was larger than for the sediment available-P (Table 2). Annual average sediment soluble-P concentrations showed similar trends across all five sites, with the highest concentration in the surface layer at 0.37 mg kg^−1^, followed by the middle layer at 0.27 mg kg^−1^, and the bottom layer at 0.17 mg kg^−1^. The variance in sediment soluble-P concentration was highest in the middle layer, at 107%, and was 83% and 76% in the surface and bottom layers, respectively. The ranges of sediment soluble-P concentrations in surface, middle, and bottom sediments were 0.10–0.83 mg kg^−1^, 0.07–0.52 mg kg^−1^, and 0.05–0.31 mg kg^−1^, respectively.

### 3.2. Spatial Variability in Sediment Available-P and Soluble-P

Annual average sediment available-P and soluble-P for the 5 representative sites are summarized in Table 3 and Table 4. Sediment available-P concentrations were significantly higher in the surface layers than the middle and bottom layers at all sites (Table 3), and the middle layers were significantly higher than the bottom layers at all but the Xinjie site. Among the sampling sites, Haigeng had the highest average concentrations of sediment available-P in all sediment layers, followed by the Luojiacun and Kunyang sites. The Dounan and Xinjie sites had the lowest sediment available-P concentrations.

Annual average soluble-P concentrations were higher in the surface layer than the middle and bottom layers at the Dounan, Xinjie, and Kunyang sites, and higher in the surface and middle layers than the bottom layer at the Haigeng and Luojiacun sites (Table 4). Concentrations of soluble-P also varied among sites. According to average soluble-P concentrations, the sites were ordered: Haigeng, Luojiacun > Kunyang > Xinjie > Dounan, which was consistent with the spatial variability in sediment available-P concentrations.

### 3.3. Variation in Sediment Available-P and Soluble-P among Sites

#### 3.3.1. Variation in Sediment Available-P

**Figure 2:**
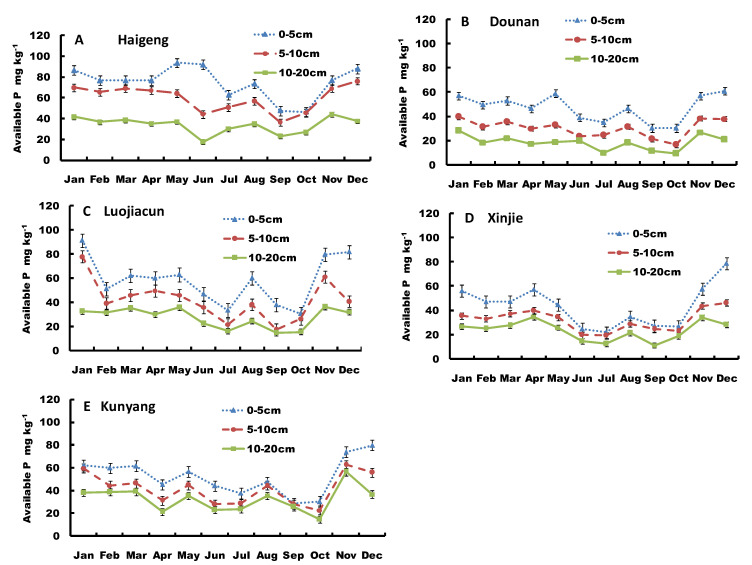
(**A**–**E**) Monthly variation in sediment available-P (S-AP) at different sites of Dianchi Lake.

The sediment available-P concentrations at different sites and different layers in different seasons are shown in Figure 2. It can be seen there was high variability in sediment available-P among different sites, different layers, and different seasons. Except for October, sediment available-P concentrations in Haigeng decreased in the order: surface > middle > bottom, which ranged within 46.4–93.6 mg kg^−1^, 36.18–76.1 mg kg^−1^, and 17.4–44.1 mg kg^−1^, respectively (Figure 2A). During the sampling year, sediment available-P concentrations were the lowest in September and October. The variation of sediment available-P concentration was higher in the bottom layer than the surface and middle layers, ranging from 21% to 23%.

Annual dynamic variation of sediment available-P concentrations in Dounan had a similar trend to Haigeng (Figure 2B), except in June, sediment available-P concentrations were in the order: surface > middle > bottom, at 30.5–60.9 mg kg^−1^, 16.7–39.7 mg kg^−1^, and 9.2–34.1 mg kg^−1^, respectively. Variation was higher in the bottom layer than in the surface and middle layers, ranging from 23% to 38%. Sediment available-P concentrations were lower between July and October than between November and June and averaged 32.4 mg kg^−1^.

Sediment available-P concentrations in Luojiacun had ranges of 30.7–91.4 mg kg^−1^ in the surface layer, 14.6–78.1 mg kg^−1^ in the middle layer, and 14.6–35.9 mg kg^−1^ in the bottom layer, decreasing in that order (Figure 2C). The variability was lower between July and August than between November and June, and higher in the middle layer than the surface and bottom layers. The variance among different layers ranged from 30% to 40%.

In the Xinjie site (Figure 2D), sediment available-P concentrations were also ordered: surface > middle > bottom, at 15.1–78.8 mg kg^−1^, 19.4–46.4 mg kg^−1^, and 11.43–34.9 mg kg^−1^, respectively. Variance in the surface sediment was higher than in the middle and bottom layers, ranging from 28% to 43%.

Figure 2E shows the annual variation in sediment available-P concentration at the Kunyang site. The sediment available-P concentrations decreased in the order: surface > middle > bottom, with ranges of 28.9–79.8 mg kg^−1^, 22.7–62.6 mg kg^−1^, and 14.8–56.6 mg kg^−1^, respectively. The available-P concentrations were lower in summer than winter. There was less variation in the bottom layer than the surface and middle layers, ranging from 31% to 35%.

#### 3.3.2. Variation in Sediment Soluble-P

**Figure 3:**
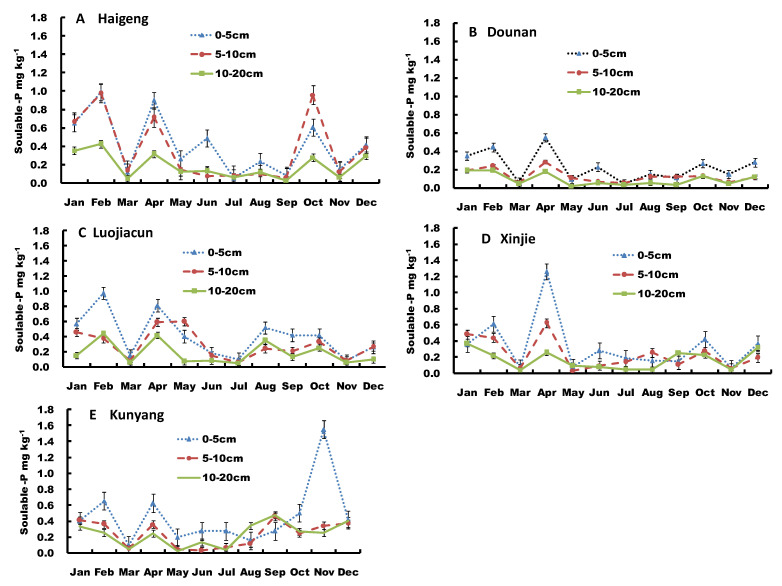
(**A**–**E**) Monthly variation in sediment soluble-P (S-SP) at different sites of Dianchi Lake.

Sediment soluble-P concentration varied among different sites, layers, and times (Figure 3). However, the annual variability in sediment soluble-P concentrations in different layers was similar among all sampling sites. The soluble-P concentrations of sediment at the sites exhibited similar trends; higher in the surface and middle layers than the bottom layer, and the difference was especially pronounced at the Haigeng, Luojiacun, and Kunyang sites. The soluble-P concentrations of the surface, middle and bottom layers were 0.08–0.99 mg kg^−1^, 0.08–0.98 mg kg^−1^, and 0.03–0.42 mg kg^−1^ for Haigeng; 0.04–0.55 mg kg^−1^, 0.05–0.28 mg kg^−1^, and 0.02–0.19 mg kg^−1^ for Dounan; 0.08–0.98 mg kg^−1^, 0–1.63 mg kg^−1^, and 0.05–0.44 mg kg^−1^ for Lujiacun; 0.08–1.26 mg kg^−1^, 0.04–0.62 mg kg^−1^, and 0.04–0.37 mg kg^−1^ for Xinjie; and 0.10–1.55 mg kg^−1^, 0.03–0.46 mg kg^−1^, and 0.02–0.48 mg kg^−1^ for Kunyang, respectively. The annual average concentrations of soluble-P were higher at the Haigeng, Luojiacun, and Kunyang sites, ranging between 0.31 and 0.32 mg kg^−1^, compared to the Xinjie and Dounan sites, which ranged between 0.25 and 0.15 mg kg^−1^.

The variation of sediment soluble-P concentrations in different layers was highest in Luojiacun, ranging from 69% to 121%, followed by 71% to 100% in Xinjie and 73% to 97% in Haigeng, with low variation in Dounan ranging between 59% and 73% and Kunyang, ranging between 63% to 86%. During the sampling year, sediment soluble-P in different layers were higher from January to May in all sites, except for the Kunyang site, where the soluble-P peaked in November in the surface sediment and in September in the middle and bottom layers.

### 3.4. Variation in Surface Sediment-P and Water-P at the Sediment–Water Interface in Dianchi Lake

#### 3.4.1. Relationship between Surface Sediment Available-P and Water Total-P

**Figure 4:**
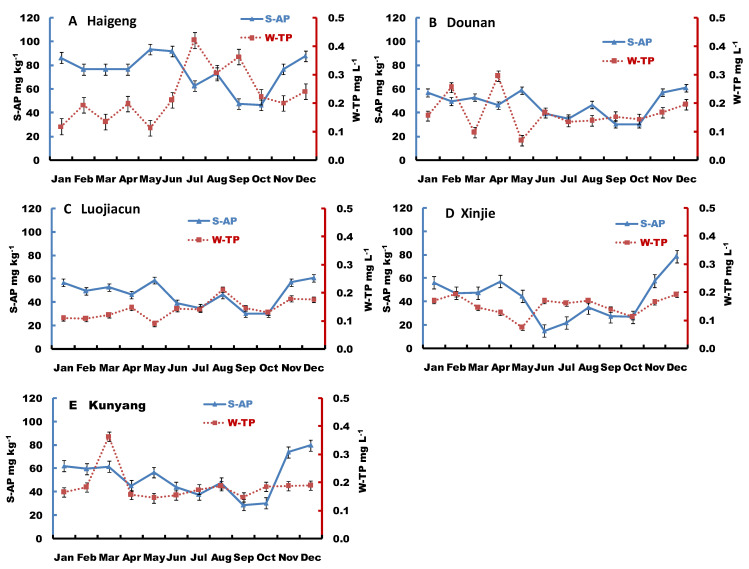
(**A**–**E**) Sediment available-P (S-AP) (0–5 cm) and water total phosphorus (W-TP) at the sediment–water interface of Dianchi Lake.

The surface sediment available-P concentration and water total-P concentration varied among different sites during the sampling season (Figure 4). There was a significant negative correlation between sediment available-P concentrations and water total-P concentrations at the Haigeng site (y (water total-P) = −0.0037x (sediment available-P) + 0.5044, r = 0.598 *, *p* < 0.05, *n* = 12 in a year, * indicates significant correlation). However, this negative correlation was not apparent at other sites. In contrast, the changes in sediment available-P concentrations showed similar trends to the those in water total-P during most of the sampling period, except in May at the Luojiacun, Xinjie, and Kunyang sites.

#### 3.4.2. Relationship between Surface Sediment Soluble-P and Water Total-P

**Figure 5:**
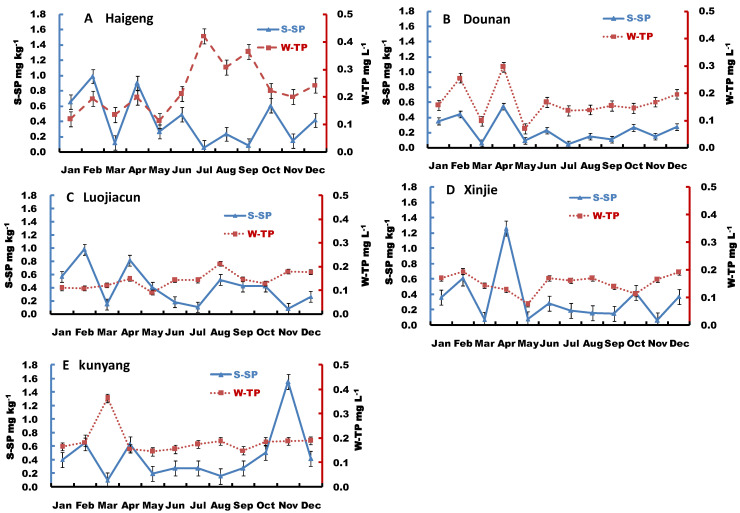
(**A**–**E**) Sediment soluble-P (S-SP) (0–5 cm) and water total phosphorus (W-TP) at the sediment–water interface of Dianchi Lake.

The relationships between sediment soluble-P concentrations and water-P concentrations at different sites were similar to the relationships between sediment available-P concentrations and water total-P (Figure 5). At the Haigeng site there was a significant negative correlation between sediment soluble-P and water total-P (y (water total-P) = −0.2372x (sediment soluble-P) + 0.3701, r = 0.836 **, *p* ≤ 0.01, *n* = 12 in a year, ** indicates a very significant correlation), and at the Dounan and Xinjie sites soluble-P concentrations were positively correlated with water total-P concentrations (y = 0.1492x + 0.1198, r = 0.772 *, *p* < 0.05, *n* = 12 in a year for Dounan; y = 0.1492x + 0.1198, r = 0.772 *, *p* < 0.05, *n* = 12 in a year for Xinjie, * indicates a significant correlation). These correlations indicated that algal growth greatly affected P movement at the interface between sediment and water at the Haigeng site, while P movement at the interface between sediment and water at the Dounan and Xijie sites was mainly affected by water disturbance. However, at the Luojiacun and Kunyang sites, sediment soluble-P was generally at a slow equilibrium with water-P.

### 3.5. Relationships between Sediment Available-P, Soluble-P, and Chlorophyll-a Concentrations in Dianchi Lake

**Figure 6:**
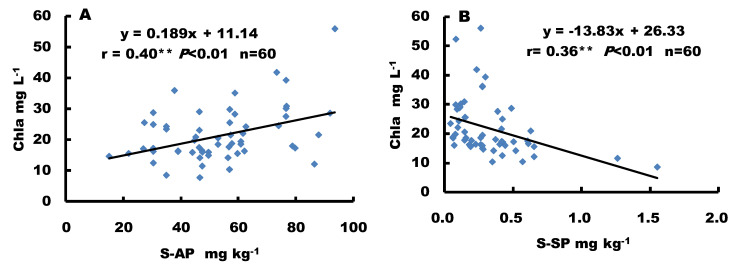
(**A**,**B**) Correlations between sediment available-P (S-AP), soluble-P (S-SP), and Chlorophyll-a (Chl-*a*). ** indicates a very significant correlation.

The sediment available-P was significantly positively correlated with Chlorophyll-a concentrations (Figure 6A; r = 0.40, *p* < 0.01, *n* = 60), but the sediment soluble-P was significantly negatively correlated with Chlorophyll-a concentrations (Figure 6B; r = 0.36, *p* < 0.01, *n* = 60).

## 4. Discussion

### 4.1. Loading of Available-P and Soluble-P in Sediments of Dianchi Lake

Sediment available-P extracted by NaHCO_3_ can be used as a quantitative index for algae available-P [11,12]. Sediments represent an important pool of available-P in freshwater systems. Available-P includes portions of organic labile-P, Fe-P, Al-P, Ca-P, and adsorbed P, all of which can be easily released into lake water in conditions of pH > 7 [12]. The available-P concentration in Dianchi Lake was high, averaging 41.2 mg kg^−1^ in 0–20 cm sediment and ranging from 9.5 mg kg^−1^ to 93.6 mg kg^−1^ during the year-long study period. Therefore, sediment available-P in Dianchi Lake is easily released when the lake water pH is 8–9. In addition, this indicated the quantity of available-P and its availability to biological organisms in the lake varied with environmental conditions and seasons, with sediment available-P acting as an important source of P for algae growth in Dianchi Lake, possibly leading to algal blooms (Table 3 and Figure 2). Indeed, there was a significant negative correlation between sediment available-P concentrations and water total-P concentrations in Haigeng site during algal blooms. The available-P concentrations in surface sediments (0–5 cm) were much higher than the nearby agricultural soils, ranging from 26.3 to 36.5 mg kg^−1^ for soils [30]. This indicated that the high P concentrations in surface sediments were related to expansion of Kunming City and the increased agricultural and industrial activities around Dianchi Lake. Anthropogenic activities associated with urbanization, agriculture, and industry can result in P enrichment in sediment. Furthermore, sediment available-P varied among seasons and sites, indicating water temperature, disturbance, microbial activity, etc., can drive the release of available-P from surface sediments into water, where it facilitates algal growth.

The available-P and soluble-P concentration in the surface sediments of Dianchi Lake were lower than those recorded in Taihu Lake in China [12], but total-P concentrations in the surface sediments of Dianchi Lake were much higher than those of Taihu Lake [26], indicating that the amount of sediment available-P released into the water in Dianchi Lake may be higher than in Taihu Lake, resulting in elevated algal growth. Soluble-P was a small fraction of the available-P in the sediment, with low concentrations ranging from 0.02 to 0.99 mg kg^−1^. The variability in soluble-P was higher than sediment available-P, indicating that soluble-P was more strongly affected by algal growth, water temperature, disturbance, microbial activities, etc., than available-P.

### 4.2. Temporal and Spatial Variability in Sediment Available-P and Soluble-P in Dianchi Lake

Variation in sediment available-P concentrations in different sites of Dianchi Lake was similar among the 0–20 cm, 20–40 cm, and 40–60 cm sediment depths with seasons. Similar patterns were also found for soluble-P concentrations. However, sediment-available-P and soluble-P concentrations in Dianchi Lake varied significantly among sites, layers, and seasons, with lower available-P occurring between July and October and higher between November and June, which was related to seasonal temperature and microbial activity changes in the lake, indicating that available-P and soluble-P load were affected by environmental and seasonal changes. Surface sediment was the main interface at which P accumulated, and was affected by P mineralization, precipitation, and adsorption. The variance in sediment soluble-P concentration with time was higher than sediment available-P concentration, especially in the middle sediment layer, we assumed that sediment soluble-P might be easily released into the water body during anaerobic conditions, especially in the middle layer (5–10 cm). Average available-P and soluble-P concentrations at different sites followed the order: Haigeng, Luojiacun > Kunyang > Xinjie > Dounan, which was consistent with the spatial distributions of algal growth (Haigeng, Luojiacun > Kunyang > Xinjie > Dounan) in Dianchi lake [25]. In addition, sediment available-P concentration was significantly correlated with Chlorophyll-a concentrations, indicating that algal growth was closely related to available-P release in lake sediment. The highest concentrations of available-P in sediment occurred at the Haigeng site where severe algal blooms occurred in summer (between July and August) and might have been related to long-term enrichment of available-P from urban and industrial wastewater discharge [31]. The Luojiacun site was located in the center of Dianchi Lake, farther from the lake shore and much deeper (7 m) than other sites (4~5 m). This facilitated the accumulation of available-P in the fine clay sediment and meant there was much less water disturbance. The Kunyang site was located near phosphate fertilizer production bases where large amounts of high P concentration wastewater were discharged into the lake, resulting in high P concentrations in both water and sediment. The phosphorus at the Dounan and Xinjie sites mainly came from agricultural non-point pollution, and the limited P loading in the sediments at these sites was more strongly affected by water disturbance compared to the Luojiacun site, resulting in lower available-P concentration in the sediments. The variance in available-P concentration was also highest at the Xinjie site. This may have been due to the seasonal P input from the rice fields and the strong water disturbances. The lowest variance of available-P was found at Haigeng, which has been observed to have a great buffering capacity and high availability of P enhanced by large amounts of organic labile-P in sediments, and the anaerobic conditions in the sediments here further help to maintain the high concentrations of available-P, with organic-P concentrations of 246~866 mg kg^−1^ [23].

Different forms of P and organic matter exhibited different distributions in the sediments of Dianchi Lake [23,32]. Due to different environmental conditions, the highest Fe-P and Al-P concentrations were in Haigeng and the highest Ca-P was in Kunyang, illustrating the variability in the amount of available-P released from sediment among sites, layers, and seasons.

In anaerobic lake sediments, organic matter is the most important factor contributing to reducing conditions, which in turn increases the bio-availability and transformation of T-P, like FePO_4_ to Fe_3_ (PO_4_)_2_, etc. The organic matter concentration in the sediments of Dianchi Lake was much higher than that of Taihu Lake [32]. Additionally, organic-P in near surface sediments can be more quickly released due to the suspended and anaerobic conditions [33], which might explain why the available-P released from sediments in Dianchi Lake was much higher than in Taihu Lake, especially at the Haigeng site with higher algae blooms in summer. The amount of sediment available-P released at the Haigeng site, due to highest Fe-P, Al-P, and organic matter, was much higher than at the other sites, resulting in the highest TP water and severe algal blooms at the Haigeng site compared to other sites.

Soluble-P is the most active and labile fraction of the available-P in sediment, and its content is directly related not only to the quantity and availability of sediment available-P, but also the water-P concentration and the other environmental conditions, like temperature, oxidation-reduction potential, microbial activity, and water disturbance. Therefore, the variation in soluble-P in sediment among the sites was even higher than that of available-P. Concentrations of available-P in sediment were low between July and August and high between November and June. In contrast, concentrations of total-P and soluble-P in water were high in summer (between July and August) and low in winter (between November and December). This indicated that release of available-P and soluble-P from sediment are directly related to water-P concentrations and affected by seasonal changes in temperature and algae growth.

### 4.3. Sediment Available-P and Soluble-P Exchange at the Sediment–Water Interface

The concentrations of available-P and soluble-P were closely related to water-P concentration. The surface sediment available-P and water total-P dynamics varied among sites during the sampling season (Figure 5). There was a significant negative correlation between sediment available-P concentrations and water total-P concentrations in the Haigeng site, which indicated that there was a dramatic and rapid exchange of P at the sediment–water interface that was directly affected by water temperature and algal growth cycle. The changes in sediment available-P concentrations showed similar trends to the those in water total-P during most of the sampling period, indicating the release of sediment available-P to the water and the replenishment of sediment available-P from water-P are very slow processes. Generally, it appeared that higher sediment available-P concentrations had a better ability to buffer changes of water total-P.

Some sediment-P can be easily desorbed and released, particularly when the concentration of phosphorus in the water column is depleted. The sediment–water interface can be anaerobic during algal bloom periods, which means biological activities may play an important role in the release and deposit of P [9], especially in the Haigeng site with higher algae blooms in summer.

Available-P concentration and properties of the sediment determine P release from sediment to the water column. However, the amounts and rates of P release, and the replenishment of available-P and soluble-P in sediment, are affected by sediment organic matter concentration, texture, water temperature, water-P concentration, etc. Therefore, the concentration of available-P in the sediment varied among sites, layers, and seasons. Some studies have indicated that the insoluble or organic-P in total-P could also be transformed or released into available-P, as sediment available-P is released into water [9]. The seasonal available-P dynamics in surface sediment were closely related to water temperature and algal growth in Dianchi Lake. Furthermore, it has been directly related to changes in various forms of water-P, especially in deeper waters [25,26]. Total-P is a very stable P pool, maintaining a certain capacity to supply P to the available-P pool, which is the most labile, readily available portion of sediment-P [33]. Therefore, high sediment total-P in Dianchi Lake could convert into available-P in summer [25]. Available-P has a high dissociation rate, permitting the rapid replenishment of water-P. However, even though the depletion of labile sediment-P can drive the transformation of nonlabile-P to labile-P, the rate is very slow. Usually, available-P (quantity factor), which includes both adsorbed-P and labile organic-P, can be released into solution by numerous chemical, physical, and biologic processes, such as dissolution, desorption, and mineralization when temperature is high and the solution P is low. On the other hand, high solution P concentration and a large organic matter deposits in the sediment can also increase sediment available-P concentration. In a lake, the actual release of phosphorus from lake sediments is governed not only by the reservoir of exchangeable phosphorus, but also other factors such as pH, redox potential, and bioturbation [15,33]. Release of P from sediment at the sediment–water interface was high in both the Haigeng and Luojiacun sites, which may have been related to their high available-P concentrations and the more suspended and anaerobic conditions that enhance P release [34,35,36]. Some sediment-P can be easily desorbed and released, particularly when the concentration of phosphorus in the water column is depleted, as was observed during the blue algal bloom at the Haigeng site of Dianchi Lake. The available-P dynamics varied among sites, but generally had significant correlations with sediment soluble-P and water total-P, which indicated that sediment available-P is important as a P supply and in driving eutrophication as a P reserve in Dianchi Lake.

### 4.4. Effect of Algal Growth on P Releases

Generally, high sediment-P and soluble-P concentrations maintained high water-P concentrations in Dianchi Lake, benefiting algal growth. The effect of algal growth on the release of P from sediments varied among different sites and over time in Dianchi Lake. Algal growth, expressed as water Chlorophyll-a concentration, increased with increasing sediment available-P concentration in Dianchi Lake, however, sediment soluble-P concentrations decreased with the increased Chlorophyll-a concentration due to algae growth uptake (Figure 6 B), which showed the close relationship between algal growth and sediment available-P and soluble-P concentration. Higher sediment available-P concentrations had a better ability to buffer changes of water total-P under algae growth. Sediment available-P may play an active role in maintaining high P concentrations in the water column when soluble-P was released into the water. Therefore, P release from sediment could support algal growth as an important nutrition source. Moreover, the aggressive algal growth and death might also affect the sediment-P release and replenishment processes. The quantity and rate of these processes are affected by both sediment and environmental factors.

## 5. Conclusions

This study confirmed that the large amounts of available-P in the sediment of Dianchi Lake are important internal P resources. However, the release of available-P to augment water-P, and its role in eutrophication, varied with both sediment and environmental conditions at different sites. The available-P concentration in the sediment generally decreased with depth. The surface sediment available-P plays an important role as a P supply at the sediment–water interface, and the quantity and rate of exchange with water-P were related to algal growth. The release and transformation characteristics of sediment available-P and soluble-P, and their relationship with algal growth, varied among sites and seasons. Therefore, it could be concluded that P release from sediment could support algal growth as an important nutrition source.

## Figures and Tables

**Table 1:** Annual variation in available-P in sediments of Dianchi Lake (P mg kg^−1^).

Summary Statistic	Surface Layer (0–5 cm)	Middle Layer (5–10 cm)	Bottom Layer (10–20 cm)
Annual average	55.1 ± 19.3	41.1 ± 15.8	27.3 ± 9.8
Annual variation	33.0~77.9	25.64~56.6	17.1~39.7
Maximum	93.6 ± 0.8	78.0 ± 9.5	56.5 ± 1.3
Minimum	15.1 ± 0.0	16.7 ± 2.2	9.5 ± 1.4
Variance	35%	38%	36%

**Table 2:** Annual variation in soluble-P in sediment of Dianchi Lake (P mg kg^−1^).

Summary Statistic	Surface Layer (0–5 cm)	Middle Layer (5–10 cm)	Bottom Layer (10–20 cm)
Annual average	0.37 ± 0.31	0.27 ± 0.28	0.17 ± 0.13
Annual variation	0.10~0.83	0.08~0.52	0.05~0.29
Maximum	0.99 ± 0.52	0.98 ± 0.26	0.48 ± 0.06
Minimum	0.06 ± 0.00	0.02 ± 0.00	0.03 ± 0.02
Variance	83%	107%	76%

**Table 3:** Spatial variation in available-P in sediment of Dianchi lake (P mg kg^−1^).

Sediment Layer	Haigeng	Dounan	Luojiacun	Xinjie	Kunyang
Surface (0–5 cm)	74.7 ± 12.5 a,α	47.1 ± 10.9 bc,α	58.2 ± 19.3 b,α	43.1 ± 18.4 c,α	52.3 ± 16.1 bc,α
Middle (5–10 cm)	59.6 ± 12.5 a,β	30.3 ± 7.4 c,β	41.7± 16.7 b,β	32.3 ± 9.1 bc,β	41.4 ± 13.5 b,αβ
Bottom (10–20 cm)	33.5 ± 7.8 a,γ	19.8 ± 7.5 c,γ	27.4 ± 8.2 ab,γ	23.6 ± 7.8 bc,γ	32.4 ± 11.2 a,γ
Average	56.0 ± 21.0	32.4 ± 14.2	42.6 ± 19.7	33.0 ± 14.7	42.1 ± 15.7

Values (means ± standard deviation, *n* = 12 for a year) followed by different letters indicate significant differences *p* < 0.05 (one-way analysis of variance) among different sediment sampling sites for the same sediment layer (a, b, and c), and among different sediment layers for the same sampling site (α, β, and γ).

**Table 4:** Spatial variation in soluble-P in sediment of Dianchi lake (P mg kg^−1^).

Sediment Layer	Haigeng	Dounan	Luojiacun	Xinjie	Kunyang
Surface (0–5 cm)	0.41 ± 0.32 a,α	0.23 ± 0.16 a,α	0.41 ± 0.28 a,α	0.34 ± 0.34 a,α	0.45 ± 0.39 a,α
Middle (5–10 cm)	0.37 ± 0.36 a,α	0.13 ± 0.07 b,α	0.36 ± 0.44 a,α	0.23 ± 0.19 ab,α	0.24 ± 0.16 ab,α
Bottom (10–20 cm)	0.19 ± 0.14 ab,α	0.09 ± 0.07 b,α	0.18 ± 0.15 ab,α	0.17 ± 0.12 ab,α	0.24 ± 0.15 a,α
Average	0.32 ± 0.30	0.15 ± 0.12	0.32 ± 0.32	0.25 ± 0.24	0.31 ± 0.27

Values (means ± standard deviation, *n* = 12 for a year) followed by different letters indicate significant differences *p* < 0.05 (one-way analysis of variance) among different sediment sampling sites for the same sediment layer (a and b), and among different sediment layers for the same sampling site (α).

## Data Availability

The data sets used in this study are available from the corresponding author on reasonable request, except in the case of data that are subject to third party restrictions.
